# A classification of pulmonary nodules by CT scan

**DOI:** 10.3332/ecancer.2012.260

**Published:** 2012-07-03

**Authors:** M Bellomi

## Abstract

This Image Report aims to briefly describe, giving some imaging examples, the radiological definition of the features of lung nodules as commonly used by radiologists. We hope thus to improve communication and mutual understanding between radiologists and clinicians.

## Introduction

A micronodule is a discrete, small, round, focal opacity in the lung parenchyma. Use of the term is most often limited to nodules with a diameter of less than 3 mm [1].

The majority of pulmonary nodules are asymptomatic and are detected by CT in the course of a lung cancer screening examination or as an occasional finding when performing a chest CT for other clinical problems.

Pulmonary nodules are classified as solid, partially solid, or non-solid (ground-grass opacities) [2].

## Solid nodule

A solid nodule completely obscures the surrounding parenchyma; it has homogenous soft-tissue attenuation and well-defined margins with normal parenchyma.

## Solid nodule with well-defined margins

This is a solid nodule with homogenous soft-tissue attenuation and well-defined margins with normal parenchyma.

## Semisolid nodule

This is a semisolid nodule consisting of both ground-glass and solid soft-tissue attenuation components. It partially obscures the surronding parenchyma.

## Ground-glass nodule (synonym, non-solid nodule)

These manifest as an area of hazy increased attenuation that does not obliterate the bronchial and vascular margins. They can be due to inflamatory changes, benign lesions, or carcinoma (often bronchioloalveolar).

They are most difficult to interpret for radiologists [3].

## Figures and Tables

**Figure 1:**
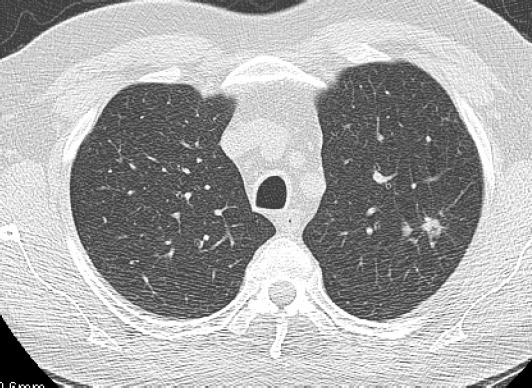
A solid nodule in the upper left lobe with irregular margins. This is a T1 adenocarcinoma of the lung.

**Figure 2:**
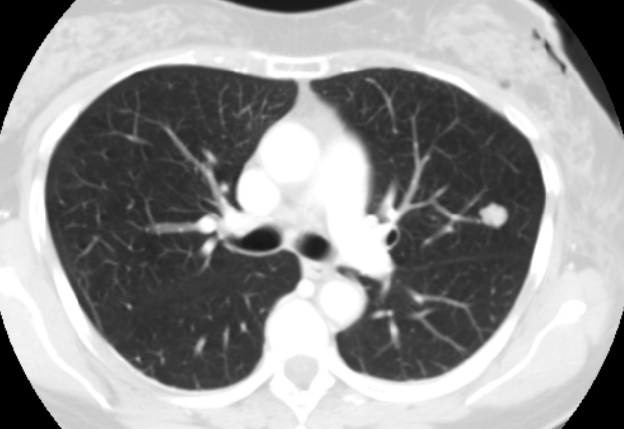
A solid nodule in the upper left lobe with regular, well-defined margins. This is a lung amartoma.

**Figure 3:**
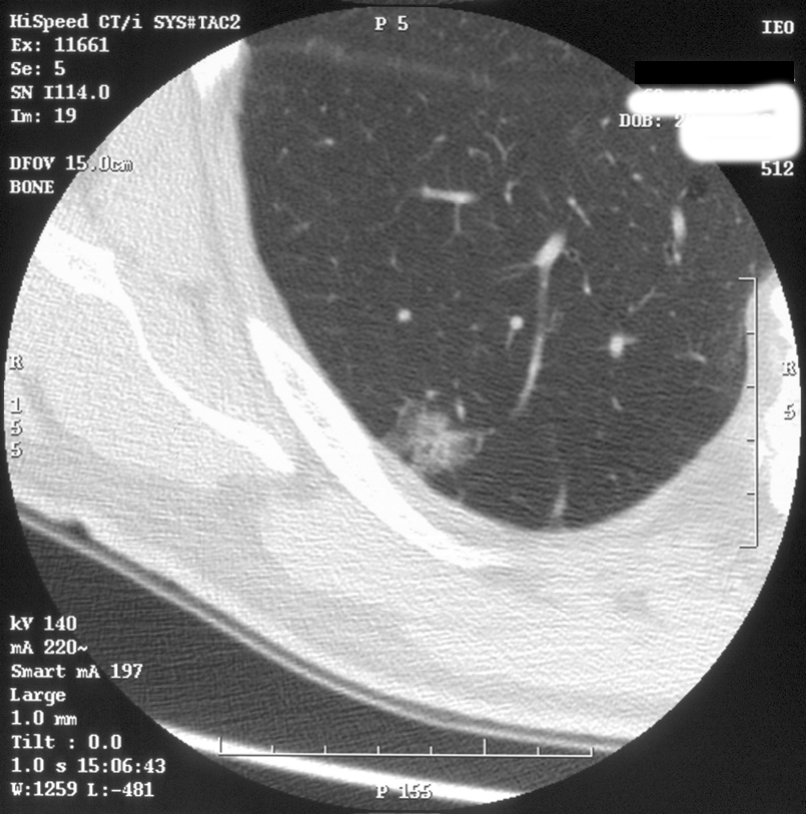
A semisolid nodule in the lower right lobe. This is an adenocarcinoma with a bronchioloalveolar component.

**Figure 4:**
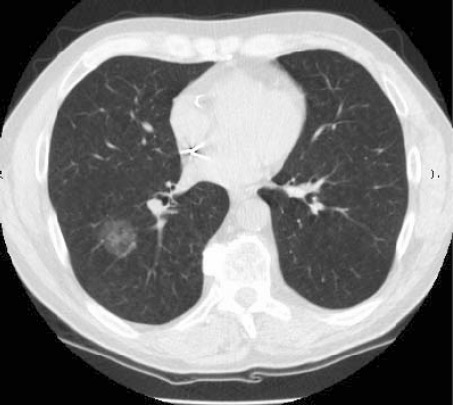
This is a non-small-cell lung cancer.
